# Development of a model for predicting mortality of breast cancer admitted to Intensive Care Unit

**DOI:** 10.4314/ahs.v22i3.18

**Published:** 2022-09

**Authors:** Renfeng Huang, Wanming Wu, Yan Guo, Linyang Ou, Xumeng Gong, Chuansheng Yang, Ruiwen Lei

**Affiliations:** 1 Department of head, neck and breast surgery, Yue Bei People's Hospital, Shaoguan, China; 2 Department of ophthalmology, Yue Bei People's Hospital, Shaoguan, China

**Keywords:** Breast neoplasms, intensive care unit, prognosis

## Abstract

**Background:**

There is still not a mortality prediction model built for breast cancer admitted to intensive care unit (ICU).

**Objectives:**

We aimed to build a prognostic model with comprehensive data achieved from eICU database.

**Methods:**

Outcome was defined as all-cause in-hospital mortality. Least absolute shrinkage and selection operator (LASSO) was conducted to select important variables which were then taken into logistic regression to build the model. Bootstrap method was then conducted for internal validation.

**Results:**

448 patients were included in this study and 79 (17.6%) died in hospital. Only 5 items were included in the model and the area under the curve (AUC) was 0.844 (95% confidence interval [CI]: 0.804–0.884). Calibration curve and Brier score (0.111, 95% CI: 0.090–0.127) showed good calibration of the model. After internal validation, corrected AUC and Brier score were 0.834 and 0.116. Decision curve analysis (DCA) also showed effective clinical use of the model. The model can be easily assessed on website of https://breastcancer123.shinyapps.io/BreastCancerICU/.

**Conclusions:**

The model derived in this study can provide an accurate prognosis for breast cancer admitted to ICU easily, which can help better clinical management.

## Introduction

Breast cancer is a common cancer in women^1^ with 1.7 million cases diagnosed worldwide each year^2^, and it is the leading cause of cancer death of female worldwide^2^,^3^. However, there were still few studies focusing on breast cancer admitted to ICU but mostly on hematological malignancies or lung cancer, and the risk factor that are associated with mortality of breast cancer is still unclear^4^,^5^. One study had shown that the most main causes of ICU admission were of cardiovascular (26%), respiratory (19%), neurologic (19%) and infectious (14%), and independent predictors of death during hospitalization were related to acute complications (like sequential organ failure assessment, cardiovascular-related admission)^6^. Another study had independently validated of APACHE II for predicting mortality of breast cancer admitted to ICU^7^.

If a breast cancer patient's risk of mortality can be graded before admitted to ICU, doctors can make a more suitable and accurate treatment plan and choose when to leave ICU. Risk score or prediction model can help stratify risk of patients but there is still not a specific prognosis model built for breast cancer admitted to ICU 8. In this study, we aimed to use comprehensive data of large cohort of breast cancer from eICU database^9^ to build a prognosis model for conveniently evaluating prognosis of breast cancer admitted to ICU, also external validate the predictive ability of APACHE IV and compared their differences. Our study is the first to develop a model for risk stratification of breast cancer admitted to ICU.

## Materials/patients and methods

### Materials and patients

This study was conducted following the ethical standards laid down in the 1964 Declaration of Helsinki and its later amendments. The data in this analysis was obtained from eICU database (https://eicu-crd.mit.edu/), which covers comprehensive conditions of patients who were admitted to ICUs throughout the United States in 2014 and 2015^9^. Requirement for individual patient consent was waived because the project did not impact clinical care and all protected health information was deidentified, which was approved by the institutional review boards of Beth Israel Deaconess Medical Center. We extracted admission data including general condition, comorbidity information, vital signs, laboratory indicators, treatment information and severity score, and clinical outcomes including in-ICU death and in-hospital death of all patients diagnosed as breast cancer into analysis. Only the first ICU admission of each patient was retained. The patients whose length of stay (LOS) in ICU was less than 24 hours; gender was not female; age was less than 18; and outcome was missed, were excluded out of the cohort. Variables with missing data of more than 35% were excluded. Vital signs were defined as mean value within 24 hours after admitted to ICU. And laboratory indicators were all defined as the maximum or minimum value of data collected within 24 hours after admitted to ICU. Treatments were all defined as operations performed within 24 hours after admitted to ICU. Comorbidity information was the diagnoses of patients in the same hospital admission. Glasgow coma scale (GCS) was the minimum value within 24 hours after admitted to ICU.

### Statistical analysis

In baseline data of patients, continuous variables were represented as median with interquartile range (IQR), compared with Wilcoxon rank-sum test. Categorical variables were represented by frequency and percentage, compared with chi-square test. General condition, comorbidity information, vital signs, laboratory indicators and treatment information from baseline data of patients except LOS of hospital, LOS of ICU and death in ICU, were used as candidate variables to develop new models. Single imputation was performed for the whole dataset based on the complete conditional specification, and predictive mean matching method was used to fill the missing value. Each incomplete variable was estimated by an independent model to ensure the validity of the imputation results 10.

Least absolute shrinkage and selection operator (LASSO)^11^ was used to screen all candidate variables and reduce the number of variables included in the model, considering the convenience of clinical application and decrease of collinearity. The model could be simplified by increasing penalty coefficients λ to compress the estimate of each variable through LASSO 12, and less variables were selected according to LASSO results and clinical significance. Afterwards, multivariate logistic regression was used to build a more concise model. The new model was then used to calculate the discrimination and calibration in the original training set. Discrimination was measured by area under the curves (AUC), and calibration was measured by calibration curve and Brier score as following formula:

$$\text{Brier score = }\frac{1}{N}\sum_{t=1}^{N}{\left(f_{t}-o_{t}\right)}^{2}$$

In the formula, N represents the total number of predictions, ft represents the actual results, and ot represents the prediction probability of the model. Then AUC, net reclassification improvement (NRI) and integrated discrimination improvement (IDI) 13 were used as indicators to compare predictive abilities of the new model and the APACHE IV scoring model. Bootstrap method was then used to validate the new model internally, and the number of repetitions was set as 1000 14. In order to evaluate the risk stratification ability of the newly built model, the study cohort had been divided into 5 groups based on the predicted probability: (1) 0–20%; (2) 20%–40%; (3) 40%–60%; (4) 60%–80%; (5) 80–100%. And the actual number of deaths and death rates were counted in each group to show whether if the model can identify the high-risk group. The clinical usefulness of the new model was then estimated with DCA by quantifying the net benefits at different threshold probabilities 15. The procedures above were all executed in R software (Version: 3.6.1). For its convenient application in clinic, we created a website according to the new model.

## Results

In the eICU database, there were totally 618 female patients diagnosed as breast cancer with 656 ICU admission records. After filtering, only 448 patients with 448 ICU admission records were enrolled in the study cohort, and 79 (17.6%) of them died in hospital. Among them, 76 (17.0%) were complicated with sepsis; 153 (34.2%) were complicated with endocrine diseases; 244 (54.5%) were complicated with circulatory diseases; 193 (43.1%) were complicated with respiratory diseases. During the data cleaning procedure, the variables with more than 35% missing data were removed. The statistical description of baseline data of study cohort is shown in Table 1.

**Table 1:** Baseline data of breast cancer 1 patients admitted to ICU

Candidate Variables		Survival Group (N = 369)	Death Group (N = 79)	P Value
General Condition				
	Age	62.00 [52.00, 73.00]	62.00 [52.50, 72.00]	0.787
	Death in ICU	0 (0.0%)	46 (58.2%)	<0.001
	LOS of ICU (hours)	58.00 [41.00, 96.00]	77.00 [50.50, 137.00]	0.002
	LOS of Hospital (hours)	143.00 [91.00, 282.00]	174.00 [98.00, 298.50]	0.512
Comorbidity				
	Sepsis	53 (14.4%)	23 (29.1%)	0.003
	Endocrine Diseases	120 (32.5%)	33 (41.8%)	0.149
	Blood Diseases	81 (22.0%)	25 (31.6%)	0.09
	Mental Diseases	52 (14.1%)	6 (7.6%)	0.169
	Circulatory Diseases	195 (52.8%)	49 (62.0%)	0.173
	Respiratory Diseases	135 (36.6%)	58 (73.4%)	<0.001
	Digestive Diseases	61 (16.5%)	16 (20.3%)	0.528
	Ischemia Heart Diseases	23 (6.2%)	1 (1.3%)	0.133
	Atrial Fibrillation Diseases	64 (17.3%)	20 (25.3%)	0.137
	Stroke	9 (2.4%)	2 (2.5%)	1
	Chronic Heart Failure	40 (10.8%)	13 (16.5%)	0.226
Vital Signs				
	heartrate (bpm)	88.03 [77.22, 101.44]	100.90 [90.61, 111.15]	<0.001
	systolic blood pressure (mmHg)	116.64 [105.31, 132.07]	109.29 [99.93, 124.82]	0.006

	diastolic blood pressure (mmHg)	62.86 [56.61, 69.28]	59.84 [53.50, 67.00]	0.176
	respiratory rate (1/min)	18.57 [16.12, 22.02]	21.00 [17.77, 24.22]	0.001
	temperature (°C)	36.84 [36.62, 37.12]	36.91 [36.56, 37.39]	0.645
	spO2 (%)	97.34 [95.91, 98.54]	96.71 [94.92, 98.00]	0.097
laboratory Indicators				
	Anion Gap ^maximum^ (mEq/L)	11.00 [9.00, 14.00]	15.90 [12.00, 17.65]	<0.001
	Anion Gap minimum (mEq/L)	9.00 [6.00, 11.38]	10.00 [8.00, 12.50]	0.021
	Bicarbonate ^maximum^ (mEq/L)	26.00 [23.00, 29.00]	24.00 [22.00, 28.00]	0.139
	Bicarbonate minimum (mEq/L)	23.00 [20.32, 27.00]	20.00 [17.00, 23.00]	<0.001
	Creatinine ^maximum^ (mg/dL)	0.84 [0.66, 1.30]	1.29 [0.80, 1.99]	<0.001
	Creatinine minimum (mg/dL)	0.74 [0.58, 1.08]	0.85 [0.62, 1.61]	0.081
	Chloride ^maximum^ (mEq/L)	105.00 [101.00, 109.00]	106.00 [102.00, 110.00]	0.297
	Chloride minimum (mEq/L)	101.00 [97.00, 105.00]	99.00 [95.00, 103.70]	0.005
	Hematocrit ^maximum^ (%)	32.90 [28.70, 37.20]	34.25 [30.80, 37.80]	0.096
	Hematocrit minimum (%)	28.80 [24.50, 33.80]	28.45 [23.67, 33.02]	0.68
	Hemoglobin ^maximum^ (g/dL)	10.80 [9.30, 12.20]	10.90 [9.60, 12.30]	0.286
	Hemoglobin minimum (g/dL)	9.50 [8.00, 11.00]	9.25 [7.60, 10.70]	0.361
	Platelet Count ^maximum^ (K/µL)	214.00 [161.00, 284.50]	196.50 [136.25, 277.50]	0.1
	Platelet Count minimum (K/µL)	185.00 [134.50, 250.50]	155.00 [69.75, 218.75]	0.002
	Potassium ^maximum^ (mEq/L)	4.20 [3.83, 4.60]	4.40 [4.10, 5.10]	<0.001
	Potassium minimum (mEq/L)	3.80 [3.30, 4.10]	3.80 [3.30, 4.10]	0.72
	Sodium ^maximum^ (mEq/L)	139.00 [136.00, 141.00]	139.00 [136.00, 144.00]	0.219
	Sodium minimum (mEq/L)	136.00 [133.00, 139.00]	134.00 [131.00, 138.00]	0.004
	Blood Urea Nitrogen ^maximum^ (mg/dL)	18.00 [12.00, 30.00]	32.00 [17.00, 51.00]	<0.001
	Blood Urea Nitrogen minimum (mg/dL)	14.50 [10.00, 23.00]	23.00 [13.00, 36.00]	<0.001
	White Blood Cell Count ^maximum^ (K/µL)	10.55 [7.68, 15.50]	13.30 [8.52, 18.85]	0.023
	White Blood Cell Count minimum (K/µL)	8.25 [5.40, 11.60]	8.40 [4.55, 13.55]	0.825
Treatment				
	Vassopressor	26 (7.0%)	21 (26.6%)	<0.001
	Anticoagulation	59 (16.0%)	8 (10.1%)	0.249
	Mechanical Ventilation	22 (6.0%)	6 (7.6%)	0.773
	Urine Output (mL)	1210.00 [760.00, 2000.00]	715.00 [329.25, 1383.00]	<0.001
Severity Score				
	APACHE IV	52.00 [40.00, 72.00]	76.50 [62.00, 93.75]	<0.001
	GCS	15.00 [13.00, 15.00]	12.00 [7.00, 15.00]	<0.001

**Figure 1:**
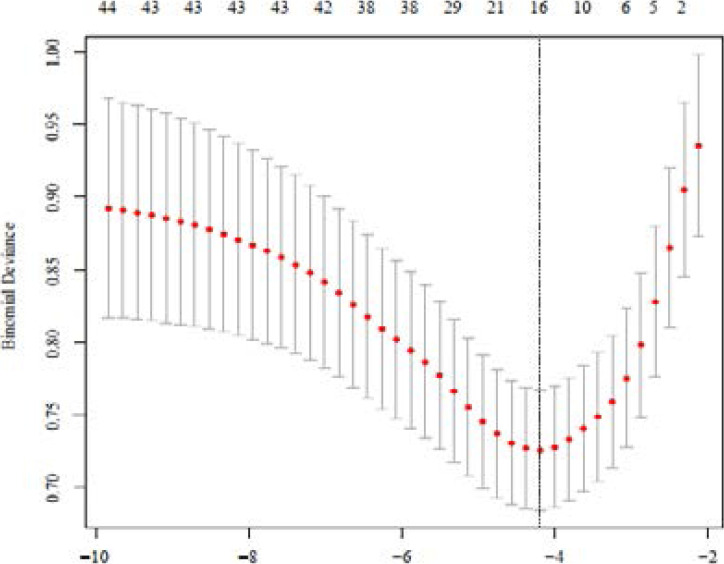
Cross-validation of logistic regression with LASSO

Then the general condition (exclude death in ICU, LOS of ICU and LOS of hospital), comorbidity information, vital signs, laboratory indicators and treatment information of baseline data were incorporated to build the model. Afterwards, LASSO was used to screen all the candidate variables of 448 patients, and tenfold cross validation for logistic regression was conducted to help select the suitable penalty coefficient λ. The relationship between λ and variables remaining in the model is shown in Figure 1.

Finally, a model composed of 5 variables (λ = 0.0678, variables including complicated with respiratory diseases, heart rate, anion gap, blood urea nitrogen and use of vasopressor) was chosen. The selected 5 variables were then put into multivariable logistic regression to fit the model. The variables and coefficients in the model are shown in Table 2.

**Table 2:** Variables 1 and coefficients in the model

Variables	Odds Ratio (95% CI)	Estimate	Standard Error	Z Value	*P* value
Intercept	-	-7.637	0.995	-7.678	-
Respiratory Diseases	4.344 (2.390–8.203)	1.469	0.313	4.691	<0.001
Heart Rate	1.027 (1.010–1.045)	0.027	0.009	3.116	0.002
Anion Gap ^maximum^	1.148 (1.079–1.225)	0.138	0.032	4.272	<0.001
Blood Urea Nitrogen ^maximum^	1.021 (1.008–1.034)	0.021	0.006	3.194	0.001
Vassopressor Use	3.356 (1.622–6.928)	1.211	0.369	3.283	0.001

Abbreviation: CI, confidence interval.

**Figure 2:**
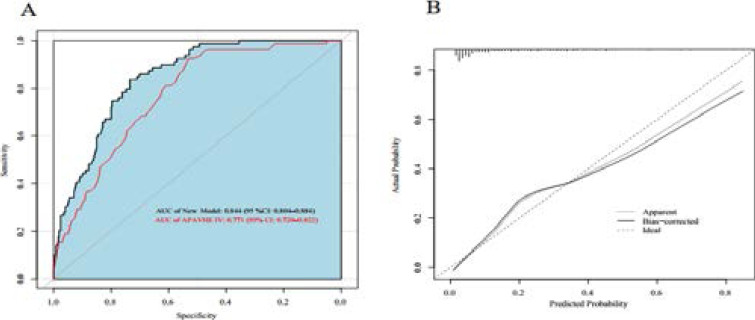
Discrimination and calibration of the model. (A) ROC curves of the new model and APACHE IV; (B) Calibration curves 3 of the new model. Calibration curve shows the mean predicted probability of outcome against the observed proportion of clinical outcomes.

All variables showed statistical significances in multivariable regression. ROC curves were then conducted to show the predictive abilities of the new model and APACHE IV, as shown in Figure 2A.

The AUC of our new model was 0.844 (95% CI: 0.804–0.884), which was higher than the APACHE IV model, whose AUC was 0.771 (95% CI: 0.720–0.822). Afterwards, the calibration of the new model was evaluated in the original training set. The calibration curve of the new model is shown in Figure 2B, and the Brier score of the model was 0.111 (95% CI: 0.090–0.127). Then bootstrap was used for internal validation, and the corrected AUC and Brier score of the new model were 0.834 and 0.116, respectively. Adjusted calibration curve is also shown in Figure 2B.

The discrimination and calibration of the new model before and after internal validation are summarized in Table 3. To evaluate the improvement of predictive abilities of the new model compared with the traditional APACHE IV model, NRI and IDI were calculated and the results are shown in Table 4. It is suggested that the predictive ability of the new model had statistically significant difference with the traditional one (both NRI [0.682, 95% CI: 0.453–0.912] and IDI [0.104, 95% CI: 0.052–0.159] were > 0, P values both <0.001), which indicated that new model had improved predictive ability compared to APACHE IV. The risk stratification ability of the model is shown in Table 5.

**Table 3:** Discrimination and calibration of models 1 in internal validation

Evaluation Index	Breast Cancer Model	APACHE IV
Discrimination		
AUC (95% CI)	0.844 (0.804–0.884)	0.771 (0.720–0.822)
Adjusted AUC a	0.834	0.770
Calibration		
Brier Score (95% CI)	0.111 (0.090–0.127)	0.126 (0.105–0.146)
Adjusted Brier Score a	0.116	0.127

a Corrected indexes were calculated with optimism bootstrap method with 1000 repetitions.

Abbreviations: AUC, area under the curve; CI, confidence interval; APACHE, acute physiology and chronic health evaluation.

**Table 4:** Improvement in prediction ability of Breast Cancer model compared with APACHE IV

	Compared to APACHE IV	P Value
NRI (Continuous) (95% CI)	0.682 (0.453–0.912)	<0.001
IDI (95% CI)	0.104 (0.052–0.159)	<0.001

Abbreviations: APACHE, acute physiology and chronic health evaluation; NRI, net reclassification improvement; IDI, integrated discrimination improvement; CI, confidence interval.

**Table 5:** Risk stratification by prediction results of the model

Prediction Result Interval	0–20%	20%–40%	40%–60%	60%–80%	80–100%
Number	310	83	32	16	7
In-Hospital Death	21 (6.8%)	30 (36.1%)	13 (40.6%)	9 (56.2%)	6 (85.7%)

**Figure 3:**
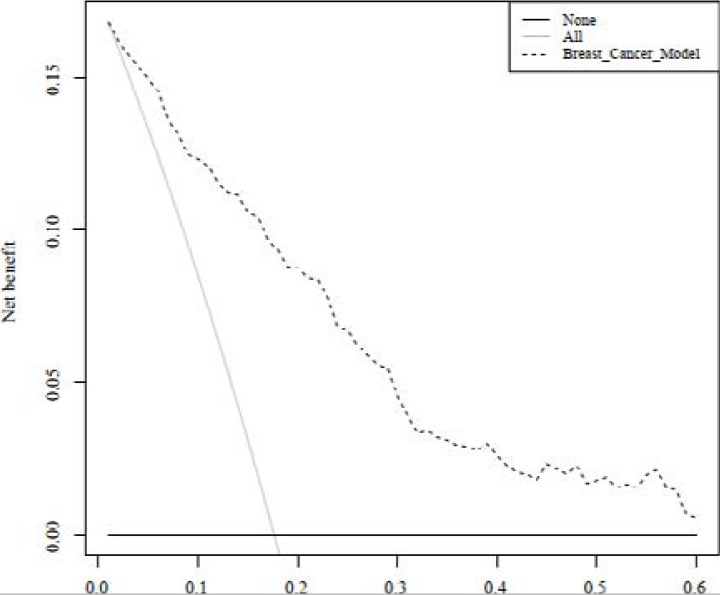
DCA for the new model

**Figure 4:**
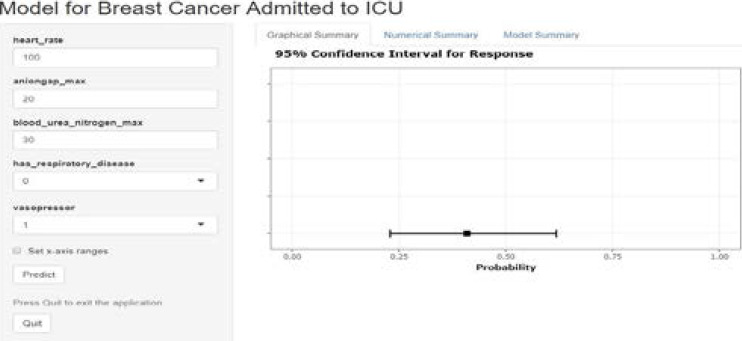
The model derived for breast cancer admitted to ICU shown in website

It was shown that model could stratified the high-risk group. The death rate was 6.8% in 0–20% group, 36.1% in 20%–40% group, 40.6% in 40%–60% group, 56.2% in 60–80% group, 85.7% in 80%–100% group. If a patient's predicted probability is higher than 40%, he/she should be paid more attention in clinic. Additionally, DCA was used to evaluate the clinical usefulness of the new model. As shown in Figure 3, the decision curve is significantly above the all-benefit line and the none-benefit line. Finally, we built a website (https://breastcancer123.shinyapps.io/BreastCancerICU/) to make our final model with 5 variables accessible in clinical application. A demonstration of the website is shown as Figure 4.

## Discussion

In this study, comprehensive data recorded in eICU database was used to develop a novel model predicting the mortality of breast cancer patients admitted to ICU with logistic regression with LASSO method. The model had been verified to have good predictive ability of in-hospital mortality of breast cancer patients, with AUCs > 0.8 both before and after internal validation. The model also had a good calibration, with a Brier score 0.111 and 0.116 before and after internal validation, respectively. In addition, the predictive ability of the model had significant improvement compared with the traditional APACHE IV model but it's more convenient to use. Besides, the model had an effective clinical use, which had been proven by DCA. The model can be easily assessed on website, which can facilitate clinical use and promote better clinical decision.

Breast cancer is the most common malignancy in women^2^. The mortality of breast cancer can be decreased these years with the progress of newer systemic anti-cancer therapies. However, cancer therapy can still lead to severe complications such as septic shock, cardiac failure or respiratory failure, and some of them need intensive care support. Previous studies had shown that the causes of admission to the ICU of breast cancer patients were mainly cardiovascular (cardiac failure, thrombosis and pulmonary embolism, syncope and arrhythmia), respiratory (pneumonia and severe respiratory distress of multi-factorial causes), neurological (seizure), electrolytic disorder, acute renal failure, and sepsis(6), and all of them are lethal complications. The ICU mortality of breast cancer patients was 15%, and the overall mortality during hospitalization was 28% (6), while the in-hospital mortality range in our study was 17.6%, and in-ICU mortality was 10.3%.

It is of significance to focus on the prognosis of breast cancer patient admitted to ICU causing if the risk of mortality can be stratified or calculated before admit to ICU, the doctors can make a more propriate treatment plan, also to decide when to leave ICU. Although ICU life support will be increasingly needed for cancer patient as assistance to accelerating treatment progress and decrease the mortality^16^, it is still a precious medical resource, which can cost a lot for patients and sometimes is not suitable for patients to stay long. For hospital and doctors, they should decide who are more urgent need of ICU admission and use this medical resource more cost-effective. Therefore, a model to evaluate prognosis of breast cancer patients with ICU admission is urgently needed for better clinical decision.

The new model contained 5 variables including 1 comorbidity information (respiratory disease), 1 vital sign (heart rate), 1 treatment (vasopressor use) and 2 laboratory indicators (anion gap and blood urea nitrogen). Complications can play an important role in prognosis of breast cancer, which has a great value to reference. Respiratory disease is a major cause for ICU admission and adverse prognostic factor for cancer patients^17^, ^18^. In our study, in-hospital mortality was 30.1% in respiratory disease group, which was higher than non-respiratory disease group (8.2%). Respiratory disease especially acute respiratory failure is a major complication of cancer patients admitted to ICU especially those who require mechanical ventilation and is a major cause for death^19^, ^20^. The most reasons for respiratory failure include pulmonary infectious, cardiogenic and noncardiogenic pulmonary edema, neoplasm-associated therapy (chemotherapy or radiation therapy) and metastasis of cancer to the lung, which are also main causes for ICU admission of cancer^21^. Previous study reported an overall survival rate of only 24% in a prospective, multicenter study of 782 adult patients with cancer receiving ventilator support for respiratory failure^22^. And a 53% survival rate was reported by another study in critically ill cancer patients with respiratory failure^23^. Other respiratory disease such as chronic obstruction pulmonary diseases and emphysema can also cause poor prognosis of cancer patients admitted to ICU^24^.

Vasopressor defined in this study including one or more use of norepinephrine, epinephrine, dopamine, phenylephrine, vasopressin or milrinone. Vasopressor is a common treatment in ICU as shock can occur in one-third of ICU admission^25^. One study had found that vasopressor therapy was appied in 61% of adult ICU patients who received anti-cancer therapy. And in hospital non-survivors, vasopressor can be occupied as 87%, which was much higher than hospital survivors (42%)^26^. In our study, in vasopressor used group, in-hospital mortality was 44.7%, which was much higher than non-vasopressor used group (14.5%), which could indicate that vasopressor was associated with adverse prognosis in breast cancer patients admitted to ICU. Vasopressor was an irreplaceable therapy to maintain an adequate mean arterial pressure in shock especially when the patients' hemodynamics is instability in ICU^27^. Shock in cancer patients can be induced by anti-cancer therapy (radiotherapy, chemotherapy or surgery), immunosuppressive, sepsis, cardiogenic or metastasis, which are all important reasons for ICU admission of cancer and can lead to poor prognosis^28^. Though vasopressor is one kind of treatment, itself also represents a critical and unstable situation of patients, which can also be used as a prognostic factor for breast cancer.

Heart rate was the only vital sign included in our model and the result had shown that a higher heart rate can cause worse outcome (OR:1.027, 95% CI: 1.010–1.045). Previous study had revealed that heart rate before ICU discharge was an independent predictor of post-ICU in-hospital mortality^29^. Another study had found heart rate >100 bpm was independently associated with increased mortality (OR 1.093, 95% CI [1.081,1.105], p < 0.001), which might differ between the elderly and non-elderly critically ill patients^30^. Two laboratory indicators including anion gap and blood urea nitrogen, were also emphasized in our predictive model. Anion gap has been also proven positively correlated to the admission rate to ICU, the persistence in ICU, and the mortality, respectively^31^. In addition, it also had been proven in our study that it is an independent prognostic indicator for breast cancer patients admitted to ICU (P <0.001) and we found that 1 mEq/L increase of anion gap improved up to 14.8% (95% CI: 7.9%–22.5%) risk of death of breast cancer. Blood urea nitrogen is also a common laboratory index which can reflect renal function, protein metabolism and even degree of heart failure condition. Blood urea nitrogen is also an important item included in multiple severity scores such as APACHE III and simplified acute physiology score (SAPS) II^32^, which can be used to predict the prognosis. Previous study also found blood urea nitrogen to be independently related with poor prognosis in critical ill patients, even after adjusted for renal failure^33^, ^34^. In all, vital signs and laboratory indicators can provide a comprehensive reference to the condition of breast cancer patients admitted to ICU and can help judge prognosis more accurately.

Our study has several strengths. First, our study had included more patients than previous similar studies into analysis and we are the first to develop a prognosis model for breast cancer admitted to ICU. Secondly, we used advanced method to build the model. LASSO belongs to machine learning and can help simplify the model while ensuring the predictive ability of the model, also to decrease the collinearity. Thirdly, our model performed more well than the APACHE IV model and can be assessed more convenient on the website.

However, our model still has some limitations. First of all, our study is a retrospective study, hence the indicators included in this study weren't sensitive enough. Some meaningful parameters might not be considered in our study such as cancer stage. Besides, in our study, the only clinical outcome in our study is in-hospital all-cause mortality. Other clinical outcomes (like discharge mortality) are not available through follow-ups as the privacy of patients were protected by the database. In addition, during data analysis and modeling, the indicators of coagulation and blood gas analysis were missing more than 35%, thus some parameters which might be clinically important were also neglected. Further studies are needed to acquire more sensitive and comprehensive data to modify the current model and overcome these limitations.

## Conclusion

We developed a prognosis model for mortality risk of critical ill breast cancer patients admitted to ICU by using comprehensive data from eICU database. A website was built to facilitate the clinical application of this model.
